# Is Bone Mineral Composition Disrupted by Organochlorines in East Greenland Polar Bears (*Ursus maritimus*)?

**DOI:** 10.1289/ehp.7293

**Published:** 2004-09-13

**Authors:** Christian Sonne, Rune Dietz, Erik W. Born, Frank F. Riget, Maja Kirkegaard, Lars Hyldstrup, Robert J. Letcher, Derek C. G. Muir

**Affiliations:** ^1^National Environmental Research Institute, Department of Arctic Environment, Roskilde, Denmark; ^2^Department of Basic Animal and Veterinary Sciences, Royal Veterinary and Agricultural University, Frederiksberg, Denmark; ^3^Greenland Institute of Natural Resources, Nuuk, Greenland, Denmark; ^4^University Hospital of Hvidovre, Hvidovre, Denmark; ^5^Great Lakes Institute for Environmental Research, University of Windsor, Windsor, Ontario, Canada; ^6^National Water Research Institute, Environment Canada, Burlington, Ontario, Canada

**Keywords:** BMD, bone mineral density, chlordane, DDT, dieldrin, endocrine disruption, osteoporosis, PCBs, polar bear, polychlorinated biphenyls, *Ursus maritimus*

## Abstract

We analyzed bone mineral density (BMD) in skulls of polar bears (*Ursus maritimus*) (*n* = 139) from East Greenland sampled during 1892–2002. Our primary goal was to detect possible changes in bone mineral content (osteopenia) due to elevated exposure to organochlorine [polychlorinated biphenyls (PCBs), dichlorodiphenyl trichloroethane (DDT) and its metabolites, chlordanes (CHLs), dieldrin, hexacyclohexanes, hexachlorobenzene] and polybrominated diphenyl ether (PBDE) compounds. To ensure that the BMD value in skull represented the mineral status of the skeletal system in general, we compared BMD values in femur and three lumbar vertebrae with skull in a subsample. We detected highly significant correlations between BMD in skull and femur (*r* = 0.99; *p* < 0.001; *n* = 13) and skull and vertebrae (*r* = 0.97; *p* < 0.001; *n* = 8). BMD in skulls sampled in the supposed pre-organochlorine/PBDE period (1892–1932) was significantly higher than that in skulls sampled in the supposed pollution period (1966–2002) for subadult females, subadult males, and adult males (all, *p* < 0.05) but not adult females (*p* = 0.94). We found a negative correlation between organochlorines and skull BMD for the sum of PCBs (∑PCB; *p* < 0.04) and ∑CHL (*p* < 0.03) in subadults and for dieldrin (*p* < 0.002) and ∑DDT (*p* < 0.02) in adult males; indications for ∑PBDE in subadults were also found (*p* = 0.06). In conclusion, the strong correlative relationships suggest that disruption of the bone mineral composition in East Greenland polar bears may have been caused by organochlorine exposure.

Bone mineral composition in mammals is based on a complex set of interrelated mechanisms and is influenced by various nutritional and environmental factors (e.g., Ganong 1991; Johansson and Melhus 2001; Johansson et al. 2002; Leder et al. 2001; Michaelsson et al. 2003; Promislow et al. 2002; Sarazin et al. 2000). Furthermore, environmental stressors such as exposure to harmful chemicals, starvation, temperature extremes, and noise have been shown to disrupt bone mineral composition in laboratory mammals (Brandt and Siegel 1978; Doyle et al. 1977; Mooney et al. 1985; Nilsson 1994; Siegel and Doyle 1975a, 1975b; Siegel et al. 1977, 1992; Siegel and Mooney 1987). The pathogenesis of stress-induced bone mineral changes is an activation of the hypophyseal–adrenal/thyroid axis, leading to enhanced parathyroid and cortisol hormone secretion, increased bone resorption, and decreased bone formation (Colborn et al. 1993; Damstra et al. 2002; Feldman 1995; Ganong 1991; Selye 1973). Other hypotheses on disruption of bone mineral status include altered mitotic rates, changes in local subcellular calcium transport, and decreased protein synthesis (Siegel and Mooney 1987).

Organochlorines such as polychlorinated biphenyls (PCBs), dichlorodiphenyl trichloroethane (DDT), chlordanes (CHLs), hexacyclohexanes (HCHs), dieldrin, hexachlorobenzene (HCB), polybrominated diphenyl ethers (PBDEs), and aryl hydrocarbon receptor (AhR)–active organochlorines (e.g., polychlorinated dibenzo-*p*-dioxins, dibenzofurans, and non-*ortho*-chlorine–substituted PCBs) are all lipophilic (low degradable) chemicals, pesticides, or unwanted chemical by-products (e.g., de March et al. 1998). In general, the presence of such compounds in the arctic marine environment is the result of long-range atmospheric transport from lower-latitude sources in more industrial areas of the world, where outputs and use of, for example, PCB peaked in the 1960s (de March et al. 1998). Because of their lipophilicity, organochlorines and PBDEs persist in the environment [Arctic Monitoring and Assessment Programme (AMAP) 2004; Colborn et al. 1993; Damstra et al. 2002; de March et al. 1998]. In polar bears, organochlorines are consequently transferred transplacentally from mother to fetus and via lactation, resulting in fetal and neonatal exposures that have the potential for adverse health effects, for example, on growth and development (Bernhoft et al. 1997; Birnbaum 1994; Polischuk et al. 1995, 2002).

In humans, PCB and DDT and its metabolites have been associated with low bone mineral density (BMD) (Alveblom et al. 2003; Beard and et al. 2000; Glynn et al. 2000) through their action as exogenous agonists and antagonists to naturally endogenous hormones (Damstra et al. 2002). Various organochlorines have also been linked to periodontitis and osteoporosis in marine fish and mammal wildlife (Bengtsson et al. 1985; Bergman et al. 1992; de Guise et al. 1995; Lind et al. 2003, 2004; Mortensen et al. 1992; Schandorff 1997; Zakharov and Yablokov 1990) and in the laboratory (Fernie et al. 2003; Jamsa et al. 2001; Lind et al. 1999, 2000a, 2000b; Render et al. 2000a, 2000b, 2001; Singh et al. 2000; Valentine and Soulé 1973). In various mammalian wildlife [e.g., gray seal (*Halichoerus grypus*), ringed seal (*Phoca hispida*), harbor seal (*Phoca vitulina*), and alligator (*Alligator mississippiensis*)], osteopenia and macroscopic pathology have been examined in bone during distinct periods of exposure to anthropogenic pollutants (Bergman et al. 1992; Lind et al. 2003, 2004; Mortensen et al. 1992, Schandorff 1997; Sonne-Hansen et al. 2002; Zakharov and Yablokov 1990). The studies showed relationships between organochlorines and exostosis, periodontitis, loss of alveolar bone structures, osteoporosis, widening of the canine opening, and enlargement of the foramen mentalia.

Polar bears from East Greenland, Svalbard, and the Kara Sea carry higher loads of organochlorines than do polar bears elsewhere in the Arctic due to the different atmospheric transport routes (AMAP 2004; de March et al. 1998; Lie et al. 2003; Norstrom et al. 1998). Subsequently, the organochlorines up-concentrate in the blubber of ringed seal (*P. hispida*) and bearded seal (*Erignathus barbatus*), which is the primary food of the polar bear (AMAP 2004; de March et al. 1998; Lie et al. 2003; Norstrom et al. 1998). Recent studies of polar bears from Svalbard have indicated that high levels of organochlorines negatively affect levels of retinol (vitamin A) and thyroid hormones (Braathen et al. 2004; Skaare et al. 2001) and possibly also negatively affect cortisols, sex steroids, and reproductive organs (female pseudohermaphrodites), although these latter mechanisms are not clearly understood (Haave et al. 2003; Oskam et al. 2003, 2004; Sonne et al., in press; Wiig et al. 1998). Other studies have associated high levels of organochlorines with low levels of IgG, suggesting possible immunotoxic effects on the IgG levels (Bernhoft et al. 2000; Lie E, Larsen HJS, Larsen S, Johansen GM, Derocher AE, Lunn NJ, et al., unpublished data). Overall, these studies support the notion that organochlorines may cause disruption and thereby potentially affect bone mineral composition.

To determine whether exposure to organochlorines and PBDEs may have adversely affected bone mineral composition in polar bears, we compared BMD in skulls of 41 individual polar bears collected in East Greenland during the supposed prepolluted period (1892–1932) with 98 polar bear skulls collected during the supposed polluted period (1966–2002). Furthermore, we examined a subset of 58 of the individuals collected during the pollution period to determine if BMD was related to body burden of various organochlorines and PBDEs.

## Materials and Methods

### Sampling and age estimation.

We studied a total of 139 East Greenland polar bear skulls sampled between Skjoldungen at 63°15′N and Danmarks Havn at 76°30′N during 1892–2002. The age determination was carried out by counting the cementum growth layer groups (GLGs) of the lower left incisor (I_3_) after decalcification, thin sectioning (14 μm), and staining (toluidine blue) using the method described by, for example, Hensel and Sorensen (1980) and Dietz et al. (1991). For analyses, the individuals were then categorized into adult males (≥6 years of age), adult females (≥5 years), and subadults (others) (e.g., Rosing-Asvid et al. 2002). Regarding skull samples from 1892–1987, the sex was available from the expedition files, and in case of absence of this information (*n* = 9), the determination was based on skull morphology.

### Osteodensitometry.

**Figure 1:**
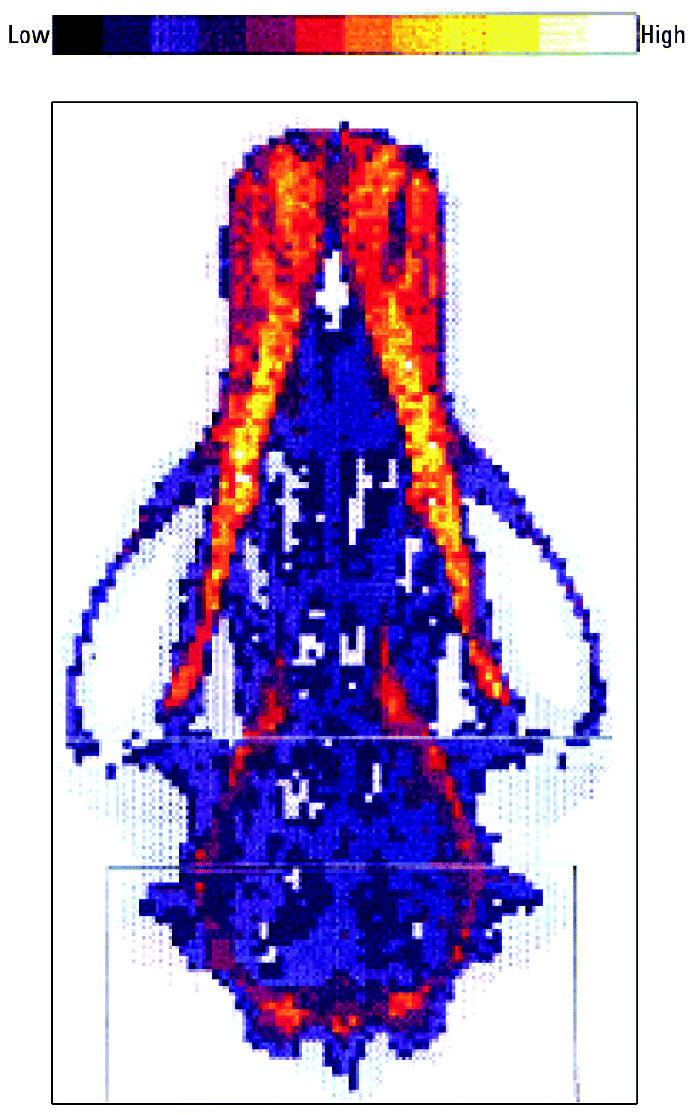
DXA scanning image of a 12-year-old female East Greenland polar bear skull sampled in 1972. Note the high-density areas of cortical bone tissue and the lower density areas of trabecular bone tissue.

X-Ray osteodensitometry was applied to detect osteopenia (osteoporosis) by use of an X-ray bone densitometer (model XR 26; Norland Corporation, Fort Atkinson, WI, USA), which determined the BMD (calcium phosphate, hydroxyapatite) using dual X-ray absorptiometry (DXA). The skulls were scanned in “research” mode (speed, 60 mm/sec; resolution, 3.0 × 3.0 mm; width, 24.9 cm) and analyzed using XR software (revision 2.4; Norland Corporation), which generated a picture of the bone segment and calculated the BMD of hydroxyapatite in grams per square centimeter (Figure 1).

To ensure that BMD in the skull represents the mineral status of the skeletal system in general, a study was conducted where the BMD of the skull, one femur, and three lumbar vertebrae were compared in a subset of 13 free-ranging polar bears (3 subadults, 2 adult females, and 8 adult males) from Svalbard and East Greenland. The DXA scanner was calibrated daily using a phantom with known mineral density. In addition, the precision was tested by a 10× rescanning (mean ± SD, 521.96 ± 0.60 g/cm^2^), which from the formula [1 − (SD/mean) × 100%] gives a precision of 99.88%. Fragmentation and loss of tooth material caused by handling and lead shot were thought to be a problem. A double determination of the BMD in 2 skulls (numbers 5483 and 2891) with and without incisors, canines, premolars, and molars showed that loss of half or more of the material of the large canines altered the result significantly. Because the canines in the material were not fragmented to such a degree, we did not consider fragmentations a problem.

### Contaminant analyses.

Polar bear subcutaneous adipose tissue samples (*n* = 58) were analyzed for PCBs, DDT and its metabolites, HCHs, CHLs, HCB, dieldrin, and PBDEs as described elsewhere (Dietz et al. 2004; Luross et al. 2002; Sandala et al. 2004). The sum of PCBs (∑PCB) is the total concentrations of the 51 individual or coeluting congeners (if detected): PCBs 31/28, 52, 49, 44, 42, 64/71, 74, 70, 66/95, 60, 101/84, 99, 97, 87, 110, 151, 149, 118, 146, 153, 105, 141, 179, 138, 158, 129/178, 182/187, 183, 128, 174, 177, 171/202/156, 200, 172, 180, 170/190, 201, 203/196, 195, 194, and 206. ∑DDT is the sum of 4,4′-DDT, 4,4′-DDD (dichlorodiphenyldichloroethane), and 4,4′-DDE (dichlorodiphenyldichloroethylene). ∑HCH is the sum of the α-, β-, and γ-hexachlorocyclohexane. ∑CHL is the total concentration of oxychlordane, *trans*-chlordane, nonachlor III (MC6), *trans*-nonachlor, *cis*-nonachlor, and heptachlor epoxide. ∑PBDE concentration is the total of 35 individual or coeluting congeners (if detected): PBDE numbers 10, 7, 11, 8, 12/13, 15, 30, 32, 28/33, 35, 37, 75, 71, 66, 47, 49, 77, 100, 119, 99, 116, 85, 155/126, 105, 154, 153, 140, 138, 166, 183, 181, and 190 (Muir DCG, Dietz R, Riget FF, Sonne C, Letcher RJ, Born EWB, unpublished data). All contaminant data are given in nanograms per gram lipid weight (l.w.).

### Statistics.

The BMD showed no deviation from normality (Shapiro-Wilk test), whereas contaminant data were log-transformed (base *e*) before analyses in order to meet the criteria of normality and homogeneity of the variance. The significance level was set to *p* ≤0.05, and a significance level of 0.05 < *p* ≤0.10 was considered a trend. First, we tested the condy-lobasal skull length versus age within each group (i.e., subadults of both sexes, adult females, and adult males) in an analysis of covariance (ANCOVA) with skull length as a dependent variable, periods (before and after 1960 respectively) as class variables, age as a covariable, and their first-order interaction links (age × period). The result from this analysis showed that the relationship of skull length versus age was the same in the two periods, which justified the use of non-length-corrected skull BMD in the further analyses (all, *p* > 0.26). Second, the relationship of BMD versus age was tested by a linear regression analyses (BMD as a dependent variable and age as an independent variable) for subadults of both sexes, adult females, and adult males. To test for period differences, we used an ANCOVA with BMD as a dependent variable, age/sex (subadult females, subadult males, adult females, and adult males) and period (before and after 1960 respectively) as class variables, age as a covariable, and the first-order interaction links (age × period, age × age/sex, and age/sex × period) between these variables. The model was successively reduced for nonsignificant interactions (*p* > 0.05) judged from the type III sum of squares, and the significance of the remaining factors was evaluated from the final model (least square means). A temporal trend over the entire period 1892–2002 was analyzed by a multiple regression analysis with skull BMD as the dependent variable and the individual age and year of kill as explanatory variables for subadults of both sexes, adult females, and adult males, respectively (the relationship was evaluated from the parameter estimate, *r*^2^, and *p*-value). The relationship between age/sex groups and contaminants was analyzed within a one-way analysis of variance on the log-transformed contaminant data, and significant results were tested by Tukey’s post hoc test. The skull BMD versus contaminant (∑PCB, ∑DDT, ∑CHL, HCB, ∑HCH, dieldrin, and ∑PBDE) relationships were explored by multiple regressions with skull BMD as the dependent variable and the age and contaminant concentrations as explanatory variables within age/sex groups (subadults of both sexes, adult females, and adult males). Finally, the relationship between levels of contaminants and BMD was evaluated from the parameter estimate, *r*^2^, and *p*-value.

## Results

We found a highly significant correlation between BMD in skull and femur (*r* = 0.99; *p* < 0.001; *n* = 13), and skull and vertebrae (*r* = 0.97; *p* < 0.001; *n* = 8). These results justified the use of BMD measurements in skull to reflect the status of the skeletal system, although information on body conditions and nutritional stressors, relevant for osteoblastic and osteoclastic activity, was not available.

### Skull BMD and age/sex differences.

**Figure 2:**
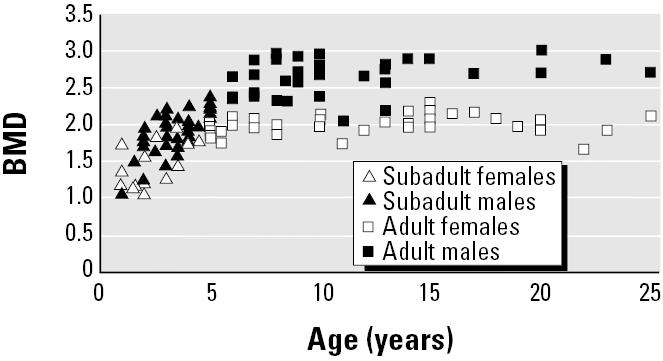
BMD (g/cm^2^) in skulls from East Greenland polar bears versus individual age.

BMD was analyzed in 139 skulls representing the period 1892–2002, and consisted of 64 subadults, 40 adult females and 35 adult males. The BMD increased with age in subadults (*p* < 0.001) but not adults (both, *p* > 0.05; Figure 2). BMD differed between males and females (*p* < 0.01) in the order subadult females < subadult males < adult females < adult males. Furthermore, BMD in females 14–23 years of age seemed to decline significantly with age (*p* < 0.04).

### Period differences and temporal trends in skull BMD.

Forty-one skulls were available from the supposed prepollution period (1892–1932) and 98 from the supposed pollution period (1966–2002) (Table 1). BMD in skulls sampled in the pollution period was significantly lower than BMD sampled in skulls from the prepollution period for subadults and adult males (*p* < 0.05) but not for adult females (*p* > 0.9) (Table 1). In addition, the multiple regression analyses of BMD versus individual age and year of kill (1892–2002) showed that BMD decreased over the entire period in adult males (*p* < 0.01), and a similar trend was found for subadults (*p* = 0.07) (Table 2). There was no BMD time trend for adult females (*p* > 0.5).

### Skull BMD and contaminants.

The range and variation of organochlorine and PBDE contaminants (nanograms per gram l.w.) in the 58 polar bears sampled during 1999–2001 are presented in Table 3. Levels of ∑DDT, dieldrin, ∑HCH, and ∑PBDE were not different between subadults, adult females, and adult males (all, *p* > 0.07). However, levels of ∑PCB were higher in adult males when compared with adult females (*p* ≤0.05). Additional information on the relationship between organochlorines and age, sex, and season in East Greenland polar bears from 1999 through 2002 has been published by Dietz et al. (2004) and Sandala et al. (2004).

BMD was found to be negatively correlated with levels of ∑PCB (*p* < 0.04) and ∑CHL in subadults (*p* < 0.03), whereas BMD was negatively correlated with ∑DDT (*p* < 0.02) and dieldrin (*p* < 0.002) in adult males (Table 4). In addition, a trend of ∑PBDE being negatively correlated to BMD in subadults was found (*p* = 0.06), whereas no significant relations were found for adult females (Table 4).

## Discussion

### BMD and age/sex differences.

The high correlation in BMD between skull and femur and vertebrae, respectively, is useful because skull samples of polar bears (and other mammals) are present at national zoological museums all over the world, which makes various time-trend bone studies possible. Our results clearly show that skull BMD increased more rapidly in subadults compared with adults, in accordance with previous studies of ringed seals from Northwest Greenland (Sonne-Hansen et al. 2002). Female polar bears usually give birth to two cubs every third year (December) and mobilize and transfer large amounts of calcium and phosphate during gestation and during the postpartum (suckling) period, which lasts up to 2 years (Ramsay and Stirling 1988). In this period, calcium is used for fetal skeletal production and maintenance of the mother’s and offspring’s calcium phosphate homeostasis (Ramsay and Stirling 1988). Because the female polar bear mobilizes these large amounts of calcium and phosphate, adult females are expected to have a lower BMD compared with adult males. Such a difference was also found in the present study. Similar differences have been found in humans (e.g., Van Langendonck et al. 2002). As suggested for humans, the marked difference in BMD between the sexes could be the result of a higher muscle mass and strength in males, leading to higher biomechanical loading of the bone. This would lead to increased bone formation through the stimulation of the mechanotransduction system in the osteocytes (Van Langendonck et al. 2002).

Earlier studies show that sufficient levels of sex steroids (estrogens and androgens) are important in the development of the human cortical bone structures in boys, girls, teenagers, adults, and the elderly (Hampson et al. 2002; Juul 2001; Leder et al. 2001; Szulc et al. 2001). On the other hand, high levels of estrogen-active substances (intrinsic, extrinsic) stimulate the expression of secondary sexual characteristics (Hampson et al. 2002; Juul 2001; Leder et al. 2001; Szulc et al. 2001). Therefore, growth delay and osteopenia (osteoporosis) have been associated with hypogonadism and lower estrogen levels in both sexes (Leder et al. 2001; Nelson 2003; Szulc et al. 2001). The age-related decrease in BMD in females in the present study was probably associated with a menopause phase after 15 years of age, but this requires a larger sample size (Figure 2) (Derocher and Stirling 1994).

### Period differences and temporal trends in skull BMD.

In both analyses of subadults of both sexes and adult males, the individuals from the prepollution period had a higher skull BMD compared with those from the polluted period. These results suggest that there is a linkage between decreased BMD for bears from the polluted period and exposure to environmental stressors compared with bears in the prepollution period. Two major environmental stressors could be linked to mineral loss in polar bear skulls: anthropogenic organochlorine compounds and PBDEs and/or climate oscillations (AMAP 2004; de March et al. 1998; Førland et al. 2002). Concentrations of, for example, ∑PCB in the adipose tissue of East Greenland polar bears have, over the last four decades, reached levels that can elicit adverse biological effects on immunologic parameters and vitamin A levels, which may be linked to the present decrease in skull BMD (stress) (AMAP 2004; de March et al. 1998). However, during the same period global warming has resulted in a reduction in the ice coverage in the East Greenland area (Comiso 2002; Rothcock et al. 1999). Although population ecology has not been studied in East Greenland, the situation is probably similar for polar bears from the Hudson Bay area in Canada (Stirling et al. 1999). A reduction of the sea ice in the Hudson Bay area has reduced the bears’ access to ringed seals, resulting in reduced body condition and lowered natality in the polar bears (Stirling et al. 1999).

Temporal differences with respect to potential effects of PCB and DDT exposure on periodontitis and osteoporosis in gray seal and harbor seal was investigated by Bergman et al. (1992), Mortensen et al. (1992), and Schandorff (1997). They found exostosis and periodontitis, often with substantial loss of alveolar bone in mandible and maxilla (osteoporosis). These changes could have been caused by hormonal imbalance potentially induced by PCBs and by DDT and its metabolites, with malformation of the calcium helix structures around the collagen matrix (DeLillis 1989). These results are further supported by the investigations of Render et al. (2000a, 2000b, 2001). However, it must be noted that the ranges of ∑PCB and ∑DDT levels in the seals were orders of magnitude higher compared with levels in the present polar bears (Blomkvist et al. 1992).

Lind et al. (2003) investigated the BMD in the male gray seals (*n* = 43) reported by Bergman et al. (1992). The method used was peripheral quantitative computed tomography, which made it possible to distinguish between cortical and trabecular bone in os mandibularis and os radius, respectively (DXA scanning used in the present study gives the average of trabecular and cortical bone density). Three sample groups of seals were compared: 1850–1955 (no pollution), 1965–1985 (high pollution), and 1986–1997 (fairly low pollution). They found that radius trabecular BMD was significantly higher in the fairly low pollution period (1986–1997) compared with the high pollution period (1965–1985), whereas mandible cortical BMD was significantly lower in the fairly low pollution period (1986–1997) compared with the no-pollution period (1850–1955). Our study of BMD in East Greenland polar bears supports the findings of Lind et al. (2003).

### BMD levels and contaminants.

Bone density expresses the bone mineral composition determined by the activity of osteoblastic bone formation and osteoclastic bone resorption, which is regulated by androgens and estrogens through cytokines (Manolagas and Jilka 1995; Manolagas et al. 1995). Studies on Svalbard have shown that PCBs may negatively influence plasma cortisol, estrogen, and testosterone levels (Haave et al. 2003; Oskam et al. 2003, 2004) and plasma retinol and thyroid hormone levels in polar bears (Braathen et al. 2004; Skaare et al. 2001). These studies all indicate that organochlorines in Svalbard polar bears (and likely also East Greenland bears, because the organohalogen compound levels are comparable) potentially affect endocrine homeostasis, which again may lead to bone mineral loss (osteoporosis). Another polar bear study from Svalbard associated high levels of organochlorines with low levels of IgG, suggesting possible immunotoxic effects (Bernhoft et al. 2000; Lie E, Larsen HJS, Larsen S, Johansen GM, Derocher AE, Lunn NJ, et al., unpublished data). This potential effect may lower the immune response and enhance stress with increased cortisol levels, which potentially affects the bone mineral composition (osteoporosis).

The present study indicated that high concentrations of ∑PCB and ∑CHL are associated with reduced skull BMD in subadults and that ∑DDT and dieldrin are associated with reduced skull BMD in adult males. These BMD relationships with ∑PCB, ∑CHL, ∑DDT, and dieldrin concentrations in subadults of both sexes and adult males may suggest endocrine-related effects (e.g., AMAP 2002; Birnbaum 1994; Damstra et al. 2002; de March et al. 1998; Lind et al. 2003, 2004). For example, PCBs and DDT and its metabolites have shown *in vitro* and *in vivo* to be weak agonists/antagonists of estrogen-receptor–mediated activity; organochlorine-mediated induction of cytochrome P450 isozyme activity can affect circulating sex hormone levels (e.g., estrogens) (Navas and Segner 1998), and this is also of relevance in the polar bear (e.g., Letcher et al. 1996). Relationships between 4,4′-DDE concentrations and BMD in humans have been reported (Beard et al. 2000; Glynn et al. 2000). Glynn et al. (2000) found significant negative correlations between 4,4′-DDE and BMD in 68 sedentary women (where concentrations are lower compared with the present polar bears) and concluded that 4,4′-DDE may also have a negative effect on BMD in men (with contaminant levels comparable with those found in the polar bears). Lind et al. (2004) investigated the relationship between DDT and its metabolites and bone composition in juvenile female American alligators (*Alligator mississippiensis*) in Lake Apopka, Florida. Compared with data from a nonpolluted reference alligator subpopulation, the tibial trabecular BMD was increased, and the authors suggested that environmental estrogenic compounds (e.g., DDT and its metabolites) disrupted the normal bone remodeling process (inhibition of osteoclast activity), which had resulted in increased BMD.

Guo et al. (1994) found that children (*n* = 25) of primiparous PCB-contaminated mothers (Yu-Cheng rice oil disease) were significantly smaller and had less total lean mass and less soft tissue mass but not lower BMD compared with a control group. The PCB levels in the children (serum) were 10.3 ng/g l.w., which is lower than the levels in polar bears in the present study. Alveblom et al. (2003) investigated the incidence of osteoporotic fractures in fishermen and their wives from the Baltic Sea (high pollution) and compared these with fishermen from the west coast of Sweden (low pollution) as controls. For vertebral fractures, there was a significantly higher incidence rate ratio for east coast (Baltic) women compared with west coast women, and a similar but nonsignificant tendency was found for men. The PCB concentration (10 congeners) was 2,000 ng/g l.w. (serum), which was significantly higher compared with the west coast population but lower compared with the range in the subcutaneous adipose tissue of East Greenland polar bears. These environmental studies support the findings of negative associations between PCBs/DDT and BMD levels in East Greenland polar bears.

In the present study, we observed a negative correlation between ∑PBDE concentrations in adipose tissue and BMD in subadults. Disturbances in thyroid function and developmental toxicity (histopathology) have been shown to be associated with PBDEs in laboratory rats (e.g., de Wit 2002) as well as in polar bears from Svalbard (Braathen et al. 2004; Skaare et al. 2001).

## Conclusions

Skull BMD increased with age in subadults and was higher in males than in females at all ages. For adult females from 14 years of age, a menopausal BMD decrease was indicated, but further examination requires a larger sample size. BMD in skulls from subadult females, subadult males, and adult males sampled in the supposed pollution period (1966–2002) was significantly lower than BMD in skulls from the period before supposed pollution with organochlorine and PBDE compounds (1892–1932). Furthermore, correlative relationships suggest that ∑PCB, ∑CHL, dieldrin, and ∑DDT exposure negatively influenced BMD in skulls from subadults of both sexes and adult males.

## Correction

In the manuscript originally published online, the years 1892–1960 and 1961–2002 were used to represent the pre- and post-organochlorine/PBDE periods, respectively. These years have been changed throughout to reflect the years in which the skulls were actually collected (1892–1932 and 1966–2002).

## Figures and Tables

**Table 1:** Skull BMD [g/cm^2^ ± SD (*n*)] for subadult and adult East Greenland polar bears from 1892 to 2002.

Period	Variable	Subadult females	Subadult males	Adult females	Adult males
1892–1932	BMD	1.67 ± 0.37 (7)	2.22 ± 0.19 (5)	1.99 ± 0.13 (9)	2.73 ± 0.21 (20)
	Age	2.6 ± 1.3 (7)	4.4 ± 1.3 (5)	12.7 ± 3.7 (9)	11.5 ± 4.5 (20)
1966–2002	BMD	1.55 ± 0.3* (17)	1.85 ± 0.32* (35)	1.98 ± 0.13 (31)	2.49 ± 0.24** (15)
	Age	2.8 ± 1 (17)	3.2 ± 1.1 (35)	12.1 ± 6.3 (31)	10.7 ± 5.5 (15)

Data are divided into two periods: 1892–1932 (supposed organochlorine and PBDE nonpolluted) and 1966–2002 (supposed organochlorine and PBDE polluted). BMD (g/cm^2^) was obtained by DXA scanning of the entire skull, and age (years) was obtained by counting the GLG of the lower I_3_ tooth.

* *p* ≤0.05 and

** *p* ≤0.01 significantly lower during 1966–2002 compared with the 1892–1932 period for the given age/sex group.

**Table 2:** Significant results from the multiple regression analyses of skull BMD versus age and year of kill in East Greenland polar bears during 1892–2002.

Age/sex group	Equation	*r*^2^	*p*_age_	*p*_yok_	No.
Subadults	BMD = 0.193 × age − 0.00254 × yok + 6.3	0.64	< 0.001	0.07*	64
Adult males	BMD = 0.014 × age − 0.00324 × yok + 8.8	0.31	0.2	< 0.01**	35

yok, year of kill. The equation is given as [BMD = *A* × age + *B* × yok + C], with BMD (g/cm^2^) as the dependent variable and age (years) and yok (1892–2002) as the explanatory variables. *A*, *B*, and *C* are specific parameter estimates; *r*^2^ is the regression coefficient of the model; *p*_age_ is the *p*-value for age; and *p*_yok_ is the *p*-value for the year of kill.

* Nonsignificant trend of BMD decline over the entire period 1892–2002 at the 0.05 < *p* ≤0.10 level.

** Significant BMD decline over the entire period 1892–2002 at the *p* ≤0.01 level.

**Table 3:** Concentrations [mean ± SD (median), ng/g l.w.] of various contaminants in subcutaneous adipose tissue of 58 East Greenland polar bears sampled during 1999–2001.

Compound	Subadults (*n* = 35)	Adult females (*n* = 14)	Adult males (*n* = 9)
∑PCB	6,597 ± 2,726 (6,089)	5,334 ± 2,150* (5,770)	8,637 ± 4,111* (8,280)
∑CHL	1,598 ± 884 (1,469)	1,379 ± 591 (1,353)	1,055 ± 517 (914)
∑DDT	392 ± 209 (376)	358 ± 149 (366)	481 ± 331 (496)
∑HCH	196 ± 68 (172)	195 ± 186 (151)	294 ± 210 (181)
Dieldrin	210 ± 100 (196)	174 ± 70 (154)	177 ± 81 (172)
HCB	99 ± 84 (70)	75 ± 82 (51)	51 ± 28 (48)
∑PBDE	62 ± 33 (53)	53 ± 17 (53)	52 ± 16 (49)

* Significant difference between adult females and males at the *p* ≤0.05 level.

**Table 4:** Significant results from the multiple regression analyses of skull BMD versus age and contaminant concentrations in East Greenland polar bears sampled during 1999–2001.

Age/sex group	Equation	*r*^2^	*p*_age_	*p*_cont_	No.
Subadults	BMD = 0.26 × age − 0.25 × [ln(∑PCB)] + 3.1	0.59	< 0.001	< 0.04**	35
	BMD = 0.24 × age − 0.19 × [ln(∑CHL)] + 2.4	0.6	< 0.001	< 0.03**	35
	BMD = 0.25 × age − 0.18 × [ln(∑PBDE)] + 1.69	0.58	< 0.001	0.06*	35
Adult males	BMD = 0.02 × age − 0.17 × [ln(∑DDT)] + 3.4	0.69	> 0.08	< 0.02**	9
	BMD = −0.005 × age − 0.37 × [ln(dieldrin)] + 4.5	0.85	0.43	< 0.002*^#^*	9

The equation is given as [BMD = *A* × age + *B* × ln(contaminant) + *C*], with BMD (g/cm^2^) as the dependent variable and age (years) and log-transformed contaminant concentration [ln(ng/g l.w.)] as the explanatory variables. *A*, *B*, and *C* are specific parameter estimates; *r*^2^ is the regression coefficient of the model; *p*_age_ is the *p*-value for age; and *p*_cont_ is the *p*-value for contaminants.

* Nonsignificant trend of a negative correlation between BMD and ln(∑PBDE) at the 0.05 < *p* ≤0.10 level.

** Significant negative correlation between BMD and organochlorine contaminant group at the *p* ≤0.05 level.

# Significant negative correlation between BMD and organochlorine contaminant group at the *p* ≤0.01 level.
